# Beyond chronic kidney disease: the diagnosis of Renal Disease in the Elderly as an unmet need. A position paper endorsed by Italian Society of Nephrology (SIN) and Italian Society of Geriatrics and Gerontology (SIGG)

**DOI:** 10.1007/s40620-019-00584-4

**Published:** 2019-01-18

**Authors:** Filippo Aucella, Andrea Corsonello, Dario Leosco, Giuliano Brunori, Loreto Gesualdo, Raffaele Antonelli-Incalzi

**Affiliations:** 1 Department of Nephrology and Dialysis, Fondazione IRCCS “Casa Sollievo della Sofferenza”, 71013 San Giovanni Rotondo, FG Italy; 2 Unit of Geriatric Pharmacoepidemiology, IRCCS INRCA, Cosenza, Italy; 3 Division of Geriatrics, Department of Translational Medical Sciences, Federico II University of Naples, Via Sergio Pansini, 5, 80131 Naples, Italy; 4 Department of Nephrology and Dialysis, Santa Chiara Hospital, Azienda Provinciale Servizi Sanitari, Trento, Italy; 5 Division of Nephrology, University “Aldo Moro”, Piazza G. Cesare n. 11, 70124 Bari, Italy; 6 Unit of Geriatrics, Campus Bio-Medico University, Rome, Italy

**Keywords:** Chronic kidney disease, Elderly, Geriatrics, Renal ageing, Renal disease

## Abstract

The dramatic increase in prevalence of chronic kidney disease (CKD) with ageing makes the recognition and correct referral of these patients of paramount relevance in order to implement interventions preventing or delaying the development of CKD complications and end-stage renal disease. Nevertheless, several issues make the diagnosis of CKD in the elderly cumbersome. Among these are age related changes in structures and functions of the kidney, which may be difficult to distinguish from CKD, and multimorbidity. Thus, symptoms, clinical findings and laboratory abnormalities should be considered as potential clues to suspect CKD and to suggest screening. Comprehensive geriatric assessment is essential to define the clinical impact of CKD on functional status and to plan treatment. Correct patient referral is very important: patients with stage 4–5 CKD, as well as those with worsening proteinuria or progressive nephropathy (i.e. eGFR reduction > 5 ml/year) should be referred to nephrologist. Renal biopsy not unfrequently may be the key diagnostic exam and should not be denied simply on the basis of age. Indeed, identifying the cause(s) of CKD is highly desirable to perform a targeted therapy against the pathogenetic mechanisms of CKD, which complement and may outperform in efficacy the general measures for CKD.

## Introduction

In the last two decades there has been a tremendous increase in the number of elderly patients with CKD and end-stage kidney disease: older adults over 65 years encompass the most rapidly growing subset of the end-stage renal disease (ESRD) population, while there was also a significant increase in the prevalence of CKD from 10.3 to 13.1% of the population, with the greatest percentage increase in those older than 70 years of age, rising from a prevalence of 37–47% [1]. The combination of high prevalence and often subclinical disease may be an overcoming problem in the next years. Accordingly, recognizing CKD and correct referral of patients are of paramount relevance in order to implement interventions preventing or delaying the development of CKD complications and end-stage renal disease (ESRD). However, several reasons make the diagnosis of CKD in the elderly cumbersome. Distinguishing age-related decline in kidney function from true CKD may be difficult, and many people with CKD are asymptomatic or have nonspecific symptoms; thus, clinical and laboratory clues are to be searched for to make a diagnosis before symptoms become severe or complications develop. Finally, CKD and its complications may significantly impact functional status and health-related quality of life among older people, and geriatric syndromes (including cognitive status, depression, disability, frailty and sarcopenia) are more and more considered in the assessment of older people with CKD [2].

Therefore, we aimed at summarizing the evidence about differences between physiologic aging of the kidney and progressive kidney disease, as well as challenges in diagnosing kidney disease among older patients. In particular, we addressed the confounding role of comorbidity and multimorbidity and diagnostic clues to be considered in clinical practice. Finally, we described the CKD diagnostic process, including patient referral, clinical assessment and use of imaging and renal biopsy, in an attempt to promote a strict collaboration between nephrologists and geriatricians in clinical management of older patients.

The content of this article represents an expert opinion shared between nephrologists and geriatricians and not the result of a formal consensus process generating guidelines.

## Renal ageing vs progressive nephropathy

Aging may variably affect structures and regulatory functions of the kidney, and these changes may increase the propensity to develop acute kidney injury (AKI) and progressive chronic kidney disease (CKD).

**Fig. 1:**
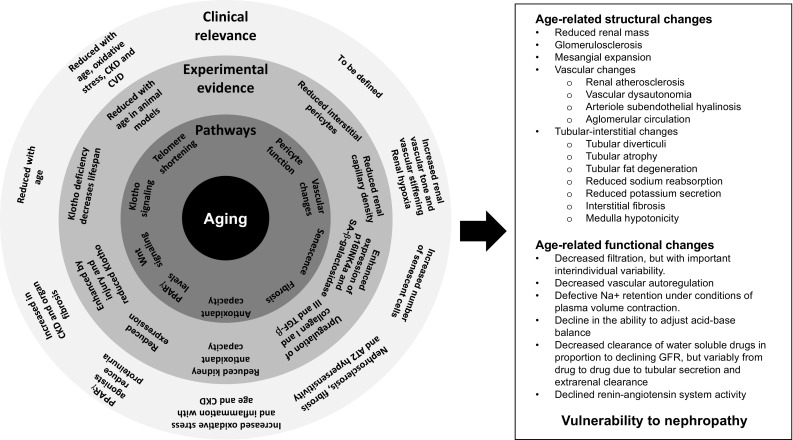
Mechanisms involved in age-related kidney function decline and their contribution to increased vulnerability to nephropathies

Overall, although renal ageing and failing kidney share some pathophysiological and clinical characteristics, very gradual changes observed in the aging kidney are clearly different from those observed in CKD, where mechanisms of progressive genetic, immune, or toxic injury are involved. Current evidence suggests that an imbalance between blunted protective factors (such as vascular density, antioxidant capacity, telomere shortening, PPARγ and Klotho expression) and stress factors (such as hypoxia, overexpression of collagen I and III, TGF-β, oxidative stress) may contribute to the activation of pathways commonly involved in kidney inflammation and fibrosis characterizing CKD. This lead to enhanced senescence and microvascular rarefaction which maintain the damage and promote progression [3]. Advanced glycation end products (AGEs) may also contribute to vascular changes in the kidney [4]. AGEs accumulate in plasma and tissues with advancing age, diabetes, and both diabetic or non-diabetic CKD [5]. Additionally, accumulation of AGEs may worsen insulin sensitivity, thus contributing to the pathophysiology of type 2 diabetes [6], and enhance tubular epithelial cells senescence [7]. These mechanisms affect kidney structures and translate into relevant functional changes (Fig. 1) [3, 8, 9].

GFR declines slowly as a function of age [10], and the rate of decline follows a fairly normal distribution, suggesting that it is primarily driven by a physiological process [11]. Interestingly, renal function remains remarkably constant in about one-third of elderly individuals: the annual average GFR reduction ranges from 0.4 to 2.6 ml/min [12, 13]. Additionally, even single-nephron GFR was found to be fairly constant with regard to age among healthy adult kidney donors aged less than 70 years [14]. The aging kidney is able to maintain fluid and electrolytes balance in normal conditions. However, the renal reserve declines and the susceptibility to acute kidney injury increases. In particular, renal hemodynamics is altered in healthy elderly due to slightly reduced GFR and significantly reduced renal plasma flow (RPF). The latter decreases more than GFR; then the filtration fraction (FF) usually increases in the elderly. Moreover, redistribution of blood flow from cortex to medulla contributes to increase FF. Additionally, due to the age-related increasing fraction of sclerotic glomeruli and the inability to increase RPF under maximal vasodilating stimuli, renal functional reserve (RFR) is reduced [15].

The aged kidney shares with the failing kidney two main functional characteristics: a low GFR and a diminished ability for tubular salt and water reabsorption. On the other hand, in healthy older people the proximal tubular function is preserved, serum levels of calcium, magnesium and phosphorus are normal, and the same is also true for serum erythropoietin and hemoglobin levels [16]. Else, erythropoietin serum levels increase with age, likely to compensate for decreased erythroid responsiveness [17].

Older people also show tubule-interstitial changes such as tubular diverticuli and atrophy, fat degeneration, interstitial fibrosis and medulla hypotonicity; both sodium reabsorption and potassium secretion are to some extent impaired [18]. In fact, both basal and stimulated secretion of renin and aldosterone are depressed as also is Na reabsorption in the thick ascending loop of Henle. On the other hand, the filtered Na is lower reflecting lower GFR. Finally, the medullary hypotonicity limits the ability to maximally concentrate or dilute the urine. As a consequence, the aged kidney takes longer to eliminate a salt load and, at the same time, less efficiently retains Na when needed. This makes the elderly at special risk of dehydration or overhydration. At variance from water and electrolyte balance, renal acidification of the urine is well preserved in the aged: following an acid load, urinary pH may be lowered efficiently, and the same is also true for titratable acid elimination [19]. However, NH_4_ elimination peaks later than in the adult in response to an acute acid load. Accordingly, plasma pH and PaCO_2_ may take longer to be restored after an acid load in the aged.

Thus, normal age-related changes in kidney function might be clinically relevant in the older patients, even in the absence of a clinically evident nephropathy, mainly in the context of conditions, such as a volume overload or a severe acidosis, impacting the renal functional reserve.

Clinicians should be able to distinguish normal ageing kidney from true renal disease (Table 1), and therapeutic efforts should focus on the patients who will benefit from interventions slowing down the progression of kidney function decline.

**Table 1:** Similarities and difference between aging kidney and CKD in regards to selected kidney function parameters

	GFR	Urea FE^a^	Urea	Ca, Mg, P FE	K FE^a^	Erythropoietin
Aging kidney	< 60 ml/m	↑	=	=	↓	=
CKD	< 60 ml/m	↑	↑	↑	↑	↓

*FE* Fractional excretion

^a^Reduced GFR may prevent full compensation by CKD-related increased urea and K FE

**Fig. 2:**
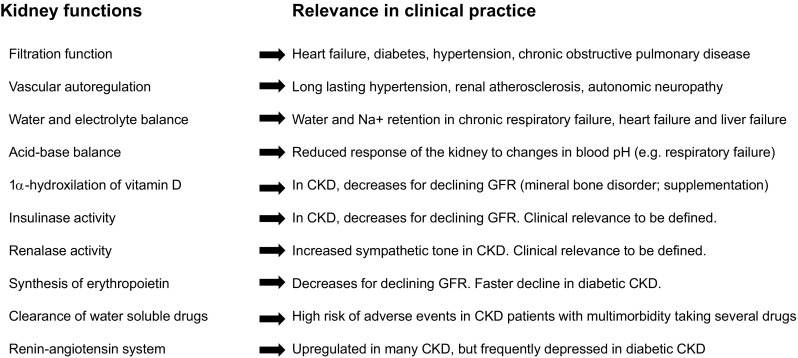
Age-related changes in kidney functions and their clinical relevance

The initial step, i.e. the diagnosis of true renal disease deserves attention and critical thinking. Reduced GFR represents the most relevant feature of kidney failure, while increased albumin-to-creatinine ratio (ACR) is a marker of kidney damage [20]. These variables represent the cornerstones of the first line assessment of patients at risk of CKD. If one or both of them are impaired, a structured search for the origin of the abnormality and to assess its severity is warranted, along with a screening for CKD complications. The presence of systemic diseases other than CKD (e.g. cardiovascular and respiratory diseases, diabetes, muscle-skeletal disorders and polypharmacy) may help the clinician to identify the cause(s) of CKD and to interpret the clinical relevance of age-related changes in kidney functions (Fig. 2).

The role of albuminuria also deserves to be mentioned. In young adults, 75% of CKD is usually characterized by albuminuria, while only 25% presents with eGFR < 60 ml/min per 1.73 m^2^. Conversely, CKD is characterized by albuminuria in only 19% of cases among older patients [21]. In healthy ageing there is a stable and minimal urine albumin excretion, while the ACR will misleadingly increase from sarcopenia of aging. However, the presence of proteinuria has clinical relevance among older patients. Indeed, the incidence of kidney failure was 3.0% among older proteinuric patients, while it was rarely observed among age-matched patients without proteinuria [22]. Thus, current evidence suggests that albuminuria/proteinuria should not be considered as a part of “normal aging” because it confers a relevant risk of worsening kidney function to older patients. On the other hand, reducing proteinuria was found to improve outcomes among older patients with IgA nephropathy [23], thus suggesting that the presence of proteinuria deserves to be carefully evaluated and monitored to limit its progression and avoid delay in the initiation of nephroprotective treatments. Conversely, low eGFR values without proteinuria may suggest to avoid renin-angiotensin system (RAS) inhibitors: patients older than age 70 years old with a GFR of 45–59 ml/min per 1.73 m^2^ without proteinuria had an estimated number needed to treat of 2500 to prevent ESRD in one patient [24]. Thus, caution is warranted in applying standard CKD treatments to a physiologic age-related decline in eGFR.

## Diagnosing chronic kidney disease in the elderly

### Confounding by comorbidity and multimorbidity

CKD usually coexists with other chronic diseases due to a pathogenetic link, e g diabetes and atherosclerosis as the cause of CKD, and to the sharing of risk factors (e.g. age and smoking habits) and pathophysiological pathways described above. Recurrent and highly prevalent clusters of diseases including CKD are the rule according to the multimorbidity approach, which defines the main associations between and among diseases without any hierarchical assumption. According to such approach, Formiga et al. [25] reported that CKD contributes to a multimorbidity cluster including coronary artery disease, hypertension, stroke and diabetes. More recently, Zemedikun et al. [26] showed that the multimorbidity cluster including CKD may be wider than that previously observed, being composed by 26 conditions, among which cardiovascular, liver, neurologic, psychiatric, respiratory, endocrine, and muscle–skeletal diseases. In these clusters, the clinical presentation of CKD may be concealed by coexistent diseases. Thus, fatigue, sarcopenia, dyspnea and anorexia may be expression of several conditions. As a consequence, CKD may remain unrecognized until blood exams performed for unrelated reasons allow detect it. Furthermore, multimorbidity accounts for polypharmacy which, in turn, is frequently nephrotoxic. The kinetics of many drugs is variously compromised in CKD, and recognizing CKD is mandatory to tailor the doses to the eGFR. Thus, renal function should systematically be screened for in the aged and multimorbid patients, and both eGFR and albuminuria should be assessed. For instance, albuminuria may address a diagnosis of nocturnal hypoxemia secondary to obstructive sleep apnea or COPD rather than a primitively renal problem [27, 28]. Furthermore, sarcopenia and hypoalbuminemia may decrease the distribution volume and the bound fraction of water soluble drugs and reduce serum creatinine out of proportion to the true eGFR, which defines the condition of concealed renal failure [29]. Indeed, diabetes mellitus, musculoskeletal diseases, diabetes mellitus and COPD are commonly associated with this condition [30].

Not unfrequently, acute kidney injury due to contrast medium unravels CKD [31–33]. Indeed, the inherent risk starts increasing from eGFR = 60 ml/min/1.73 m^2^ and further increases in patients given metformin or potentially nephrotoxic drugs as well as in those having anemia, diabetes mellitus, CHF and hypotension, which clearly are age-related conditions [34].

Noteworthy, CKD occurs in 26–53% of patients with heart failure with preserved ejection fraction (HFpEF) [35] and worsening renal function during HFpEF-related hospitalization is associated with a 7-year survival rate of only 9% [36]. It is unclear whether CKD represents a complication of other comorbidities and accounts for myocardial inflammation and fibrosis characterizing HFpEF or, instead, HFpEF causes CKD by triggering renin-angiotensin pathway activation and inducing venous congestion.

### Searching for diagnostic clues

With the many caveats due to confounding by multimorbidity, symptoms are the main clues to suspect CKD, but biological clues are also worthy of consideration. Indeed, the kidney has a primary endocrine and paracrine role besides being the main regulator of the water and electrolyte balance. As a consequence, a new onset hypertension or an important increase in the requirement of antihypertensive drugs may reflect a renal dysfunction, and sympathetic overtone may be secondary to decreased renalase activity. Water retention, with its clinical (dyspnea, edema) and laboratory or instrumental manifestations (mild hyponatremia, blood flow redistribution on the chest X rays, decreased inspiratory collapse of the caval vein at the ultrasonographic examination, findings at the bio-impedance analysis) may be a further clue to CKD. However, CKD may occasionally associate with dehydration in the context of decreased responsiveness to ADH and depressed thirst. Analogously, anemia without a demonstrable deficit of hemopoietic factors may suggest CKD, not only in stage IV, but even in stage III, given that, mainly in diabetic CKD, the renal synthesis of erythropoietin may decline faster than expected on the basis of eGFR [37]. Furthermore, an abrupt decrease in the insulin requirements may reflect depressed renal insulinase activity in severe CKD. Deficit in the α1-hydroxilation of vitamin D usually occurs in stage IV CKD [38], but other tubular dysfunction may manifest much earlier. This is the case of hyperkalemia secondary to depressed renin-angiotensin system in selected diabetic patients, of hypotension secondary to acquired defective responsiveness to antidiuretic hormones, e g after severe hydronephrosis, of hypokalemia due to potassium losing tubulopathy, which may be induced by antibiotic and antineoplastic drugs. Osteoporosis and sarcopenia may also orient towards CKD.

On these bases, a systematic screening of renal function is mandatory in a variety of clinical and laboratory conditions (Table 2).

**Table 2:** Clinical and laboratory findings suggesting CKD, but highly exposed to the confounding by and shared with coexistent chronic diseases in the elderly

Clinical clues	Laboratory clues
Fatigue, dyspnea, tachypnea	Anemia
Water retention	Hypo- or hypercalcemia
Anorexia, nausea	Hyperkalemia or hypokalemia (tubular dysfunction)
Sleep disorders	Metabolic acidosis
Difficult to control hypertension	Hypocapnia (hyperventilation compensating for metabolic acidosis)
Osteoporosis	Hyposthenuria (low urine osmolarity: nephrogenic diabetes?)
Arterial calcifications	Oliguria or polyuria
ADRs to normally dosed kidney cleared drugs	Proteinuria or hematuria
Unexplained peripheral neuropathy	Hyperchloremia: renal metabolic acidosis?
Itching	Hyperphosphatemia
Gastrointestinal bleeding	Hyperuricemia
Weight loss	Increased ESR and/or CRP
In diabetic patient: reduced cumulative amount of insulin	Increased D-dimer

Furthermore, it is highly recommended also in the asymptomatic and healthy elderly because kidney function relies upon a huge functional reserve which is consistent with well being until a critical threshold deficit is achieved. However, it is unclear how frequently renal function should be checked for in this subject. Based on the age-related yearly risk of new onset CKD, a yearly check seems recommendable, but a widespread consensus on this topic is lacking.

The screening of renal function includes the measurement of eGFR and albuminuria.

KDIGO still recommend the CKD-EPI equation [39] to estimate GFR. There is however an ongoing debate in the literature regarding the potential superiority of other equations (and other biomarkers like cystatin C), notably equations tailored to the elderly population [Berlin Initiative Study (BIS) and Full Age Spectrum (FAS)] [40–43].

Furthermore, the widespread use of CKD-EPI in the adult population allows compare different populations and observe very long-term trends. The albuminuria may be assessed by single spot analysis and, only in the event of a positive result, by confirmatory assessment of the 24 h proteinuria.

### The rationale for an active diagnostic strategy

Identifying CKD allows treat it, and the interventions are effective at any age. This is true either for causal therapy or for the overall therapy of CKD [44]. Interestingly, many treatable conditions underlying CKD are recognized in the elderly population provided that they are carefully searched for (Table 3).

**Table 3:** The most common clinical conditions underlying CKD among older people

Diabetes
Hypertension
Glomerulonephritis
Cystic/hereditary disorders
Infectious diseases
Neoplasms
Obstructive disorders
Autoimmune diseases
Hepato-renal failure
Scleroderma myeloma
Amyloidosis
Drug toxicity

Thus, even in the very old CKD should not a priori considered as the effect of vascular and/or metabolic diseases. Furthermore, many other interventions are made possible by the awareness of CKD.

- Optimizing the choice of drugs and tailoring their doses to the renal function. The high incidence of ADRs to water soluble drugs in older patients with concealed CKD and the proved effectiveness of dedicated prescribing strategies found this measure.
- Choosing the antihypertensive treatment in order to preferentially contrast the sympathetic overtone or the water retention, as indicated on individual bases.
- Downsizing the insulin therapy in severe CKD.
- Providing erythropoietin and/or D3 vitamin, if needed.
- Planning interventions aimed to improve nutritional status and provide individualized diet regimens.
- Providing alkalinizing therapy in tubular acidosis.
- Providing effective strategies for preventing contrast induced nephropaty in at risk patients [45, 46].
- Replacing B12 vitamin, if needed, through methyl and hydroxocobalamin and not through cyanocobalamin due the potential rise in serum cyanide in CKD [47]. Indeed, cyanide catalyzes oxidation of LDL cholesterol and consumes hydrogen sulfide which is a key component of endothelial derived relaxing factor [48].
- Planning and performing the immunization against selected viruses and bacteria. Given CKD is associated with immune depression, immunization against Hepatitis B virus should be provided in the early stages of CKD and, then, repeated in the end stage [49].

Overall, there is circumstantial evidence that a comprehensive approach to CKD can improve both survival and quality of life in aged populations [50].

## Stepwise diagnostic process of renal disease in elderly people

### Patient referral

When evaluating the epidemiology of kidney diseases in geriatric population, at least three main selection bias should be considered:

- the lack of overt symptoms and/or misleading laboratory parameters;
- elderly patients showing early signs of renal disease are rarely referred to a Nephrologist;
- elderly patients under the Nephrologist’s care rarely undergo a renal biopsy.

Many nephrological conditions are clinically silent up to the onset of late complications, especially in the elderly. On the other hand, very frequently abnormal laboratory findings remain neglected and do not induce the general practitioner to ask for a nephrological opinion [51]. Not unfrequently, the diagnosis and categorization of CKD relies upon serum creatinine and not on the recommended GFR estimated by common formulas [52, 53]. On the other hand, early identification and referral of people with CKD has the potential to reverse, delay, or prevent progression of disease and is a key focus of international initiatives in the area of kidney disease [54].

Obviously, as recently outlined by a joint initiative of European nephrologist and geriatrician [55], not all older patients with an eGFR < 60 ml/min/1.73 m^2^ should be labeled as having kidney disease, since this may be part of physiological aging. The universality of eGFR thresholds for diagnosis of CKD independent of age is still matter of debate. The lack of an age calibration for CKD diagnosis has been considered as an inherent and potentially serious weakness that might lead to substantial over-diagnosis of CKD in otherwise healthy populations of older subjects [56]. The most common CKD category observed in community-based programs is stage 3A (ie, GFR of 45–59 ml/min/1.73 m^2^), which predominantly affects older persons and is seldom progressive in the absence of significant proteinuria [56]. Moreover, in the elderly, a low creatinine excretion rate could contribute to an increased ACR even if total albumin excretion is close to normal and lead to classification errors between the A1 and A2 categories. An age-adjusted definition of CKD based on eGFR less than 45 ml/min/1.73 m^2^ has been proposed for older patients, but it would only be applicable to those who do not have any other corroborating signs of kidney disease such as proteinuria [57]. However, the KDIGO guidelines do not support the use of age-dependent thresholds for CKD diagnosis [51]. Indeed, evidence coming from large longitudinal studies suggest that the thresholds of low eGFR and high albuminuria are generally appropriate to define CKD across all age subgroups in terms of patient survival, and that eGFR and albuminuria should be added in the cardiovascular risk charts for the general population where only traditional risk factors are considered so far [58]. Additionally, even for older patients with stage 3a CKD computing eGFR is important for adjusting doses of kidney cleared medications. Older CKD patients more likely to benefit from closer nephrological follow-up might be identified based on the likelihood of progression of CKD and the probability of survival to end-stage [55]. In general, older patients with advanced CKD (eGFR < 45 ml/min/1.73 m^2^) should be screened regularly for functional impairment and malnutrition to identify those needing an in-depth assessment and intervention.

As regards the second selection bias, main criteria for nephrologist referral are reported in Table 4. Referral to nephrologists was found to depend upon patient’s age, being observed in 58% of patients aged less that 60 and only 11% among those aged 60 years or more [59]. More recently, an Italian study showed that the risk of incident ESRD among patients with CKD stages 3b–5 not referred to nephrology is higher than that of mortality, and selected CKD risk factors such as hypertension, anemia, and albuminuria, are associated with greater incidence of adverse events independent of eGFR level [60]. In another study, the elderly patients kept under regular surveillance in the nephrology clinic had more severe renal impairment (i.e. lower median eGFR at referral) and were more likely to have a rapid decline in kidney function compared to those discharged back to primary care [61]. This suggests that they had been correctly identified as needing specialist renal care to manage complications of CKD and eventually undergo renal replacement therapy [61]. However, considering the bulk of evidence showing that older patients with CKD exhibit an increased risk of ESRD starting from stage 3b [22, 62–64], it would be sensible considering referral to nephrologist for patients with eGFR < 45 ml/min/1.73 m^2^.

**Table 4:** Main criteria for referral to nephrologist

Hematuria (not of urologic origin)
Rapid increase in proteinuria/microalbuminuria
Proteinuria > 1g/24 h
Nephrotic syndrome
Recurrent hyperkalemia
Metabolic acidosis
Rapid progression of CDK (defined as decline in eGFR > 5 ml/m/year)
eGFR < 30 ml/min/1.73 m^2^

CKD regression may occur in one-fourth of patients receiving outpatient nephrology care, especially in the absence of high proteinuria, hypertension and diagnosis of CKD [65]. CKD regressors exhibit lower risk of ESRD and no higher risk of death. Finally, also in elderly patients who initiated dialysis treatment, timely referral, at least 1 year before initiating RRT, is associated with reduced mortality [66].

### Clinical assessment: history, physical examination, laboratory investigations

A careful history taking is mandatory to acquire the clinically relevant information. Informal support may be very useful [37]. Medication review represents an important step of clinical assessment, especially in the context of multimorbidity and polypharmacy. Potentially nephrotoxic drugs should be accurately scrutinized and eventually shifted to less nephrotoxic alternatives whenever possible. Physical examination is more time consuming and requires more patience, looking signs more likely to be present in an elderly patient, such as signs of skin infection, vasculitis, diabetic foot, enlarged urinary bladder or paraumbilical vascular bruit.

BMI should not be considered a universally reliable index of nutritional status and the measurement of arterial blood pressure may be biased by arrhythmias and vascular rigidity.

Besides increasing the risk of ESRD, morbidity and mortality, CKD also affects outcomes relevant to older people, including subjective physical function, limitations in activities of daily living (ADL) and worsening disability, impaired objective measures of physical performance, frailty, cognitive impairment, depression, impaired sensory function, nutritional disorders and sarcopenia [67].

The systematic use of comprehensive geriatric assessment (CGA) allows to identify medical, psychosocial, and functional limitations of a frail older person in order to develop a coordinated plan to maximize overall health with aging [68], and both geriatricians and nephrologists are more and more aware of the need for integrated care in this setting [69–71].

Laboratory investigations for kidney diseases do not change with age. While specific eGFR equations have been developed for older people [52, 53], the use of CKD-EPI is currently recommended [72]. Malnutrition, highly prevalent in the elderly, makes serum creatinine unreliable as a renal function marker; it may also lower other laboratory parameters such as albumin, complement C3 factor, cholesterol and lymphocyte count, which may confound diagnosis of nephrotic syndrome or nephritis where they would be expected to be increased; serum protein electrophoresis is often abnormal due to high frequency of monoclonal component requiring immunofixation of both serum and urine. Urine analysis is a key exam, as also are quantitation of proteinuria and urine culture [37].

Although urine abnormalities are the most representative findings in geriatric nephrology, they are still now the least investigated. Isolated hematuria is frequently due to urological conditions, but it may also reflect a renal disease such as thin basement membrane nephropathy or papillary necrosis. On the other hand, isolated proteinuria is never due to ageing kidney, but it may be the first sign of renal damage induced by diabetes, hypertension and so on [73].

### Imaging techniques

Two main reasons clearly support the use of ultrasonography (US) in the geriatric field: lack of potentially nephrotoxic contrast media and ease of use in geriatric common conditions. Thus, US currently qualifies as a routine investigation of obstructive nephropathy, renal mass, malignancies.

In elderly patients, both renal function and kidney volume are often reduced [74]. As intrarenal vascular compliance diminishes and subclinical chronic ischemia ensues, the kidneys become smaller. Intraparenchimal resistive index has been proposed as a way to measure nephroangiosclerosis. It has also been shown to be useful in identifying patients with preclinical hypertensive renal damage, characterized by reduced kidney volume and increased renovascular stiffness [75], as well as typical signs of renal amyloidosis [76]. Finally, Doppler sonography is of paramount relevance when investigating renal vascular disease [77].

### Renal biopsy

The overall rate of native kidney biopsy varies throughout the world, from over 250 pmp in Australia to less than 75 pmp in the United States [78], being higher in adults than in children. However, these differences do not reflect variability in the spectrum of renal pathology, but rather in the view of the diagnostic potential of biopsy. In fact, although the utility of the biopsy may differ considerably based upon the indication, the results of the renal biopsy impact patient care in up to 60% of cases [78, 79]. In recent years, renal biopsy has been underused due to the belief that in older adults histology does not add to a clinical diagnosis, except for distinctly rare glomerular diseases, and might be dangerous. Nowadays, there is consensus of opinion that older age is not a contraindication to renal biopsy [80], which is safe and reveal unanticipated diagnoses in 15–33% of cases in the elderly population (over age 60–65 years) [80, 81]. Even among the very old (over age 80 years), renal biopsy may provide valuable diagnostic and prognostic data [82].

At least two main studies focused on the risk of a misdiagnosis carried by a pure clinical diagnostic path. The former showed that only the histological diagnosis allowed the patients to be properly treated and to be protected from unnecessary and potentially harmful therapy [83]. The latter was a confirmatory study in clinically diagnosed diabetic nephropathy [84]. Thus, histology is essential to precisely characterize the glomerular diseases in the context of nonspecific clinical pictures and to direct the best therapeutic strategies.

Finally, glomerular diseases are far from being rare in advanced age, and it is important to define them [85]: incidence of primary GN is 25.7 cases PMP in the whole population, 24.8 cases PMP in people under 65 years and 30.8 cases PMP in people over 65 years, and similar findings were consistently reported [82, 86, 87]. The reluctance to diagnose elderly patients’ Glomerulonephritis extends to its treatment, and this is a further clinical bias. For example, evidence was provided that the treatment of Membranous Nephropathy [88], Focal-Segmental Glomerulo-Sclerosis and in Vasculitis [89], all are effective in elderly patients as in younger ones. Overall, immunosuppressive therapy in the elderly is effective for preventing ESRD and reducing mortality, and older age did not appear to contraindicate it [90, 91].

**Fig. 3:**
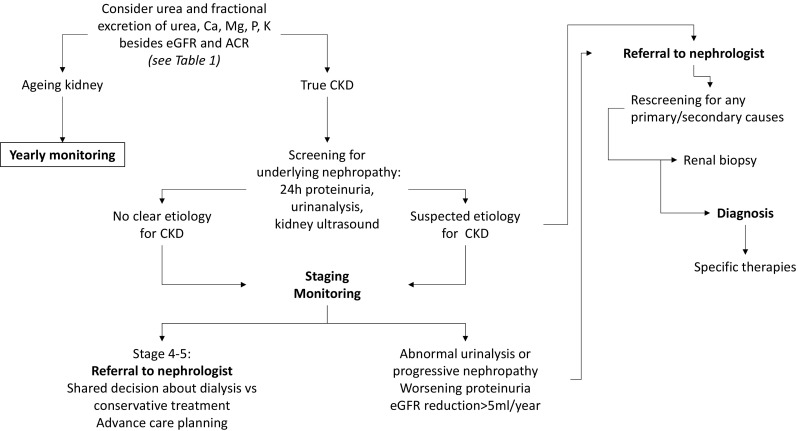
Suggested diagnostic workflow to distinguish aging kidney from CKD and to investigate underlying nephropathy

In conclusion, histology is essential to precisely characterize the Glomerular Diseases with nonspecific clinical pictures, and to optimize the therapeutic strategies, also in elderly. Elderly patients should be offered the same chance of therapy that adult patients are usually given, and the higher rate of drug side-effects should not deter the treatment. A closer monitoring of the therapy is more suitable in this setting. The role of renal biopsy in the diagnostic workflow aimed at distinguishing normal aging of the kidney from CKD and at characterizing CKD is proposed in Fig. 3. Abnormal urinalysis or progressive nephropathy, worsening proteinuria and/or eGFR reduction > 5 ml/year should be considered as relevant reasons to refer the patient to nephrologist and eventually scheduling for biopsy after re-screening for primary/secondary nephropathies. Nevertheless, it must be recognized that the level of evidence about renal biopsy among older patients remains relatively low. Thus, much more work is needed before drawing population-specific guidelines recommendations.

### Closing remarks: an integrated nephrogeriatric approach to the renal elderly patient

Nephrologists and geriatricians are more and more involved in diagnosing and caring older patients with CKD. Sharing diagnostic methodologies is particularly relevant when guidelines recommendations are lacking, as is the case of older people. Renal diseases are a major problem in the elderly, but also a treatable and, to some extent, preventable one. The complexity characterizing the elderly patient could mask kidney disease or account for several modifiable factors affecting kidney function. Thus, recognizing the renal disease and its causes as well as planning a tailored treatment requires an in depth knowledge of the patient, which frequently is out of the reach of the traditional nephrologic approach. Involving primary care physicians may help to obtain early identification and correct referral of older CKD patients. Furthermore, providing a comprehensive care of the health status implies that its many components and determinants be properly recognized. All this makes a truly multidimensional approach to this kind of patient mandatory. A strict cooperation between nephrologists and geriatricians is needed to select the best tools for the comprehensive geriatric assessment and to provide practical therapeutic recommendations. Indeed, a nihilistic attitude would carry the risk of leaving a treatable condition untreated and, then, prone to deterioration. On the other hand, a too aggressive and untailored approach might be harmful. Thus, a renewed diagnostic and therapeutic process is expected to stem from a positive and reciprocal contamination of the nephrological and geriatric cultures.

## References

[1] Collins AJ, Foley RN, Chavers B (2012). ‘United States Renal Data System 2011 annual data report: atlas of chronic kidney disease and end-stage renal disease in the United States. Am J Kidney Dis.

[2] Webster AC, Nagler EV, Morton RL, Masson P (2017). Chronic kidney disease. Lancet.

[3] O’Sullivan ED, Hughes J, Ferenbach DA (2017). Renal aging: causes and consequences. J Am Soc Nephrol.

[4] Koschinsky T, He CJ, Mitsuhashi T (1997). Orally absorbed reactive glycation products (glycotoxins): an environmental risk factor in diabetic nephropathy. Proc Natl Acad Sci USA.

[5] Henle T, Miyata T (2003). Advanced glycation end products in uremia. Adv Ren Replace Ther.

[6] Uribarri J, Cai W, Ramdas M (2011). Restriction of advanced glycation end products improves insulin resistance in human type 2 diabetes: potential role of AGER1 and SIRT1. Diabetes Care.

[7] Liu J, Huang K, Cai GY (2014). Receptor for advanced glycation end-products promotes premature senescence of proximal tubular epithelial cells via activation of endoplasmic reticulum stress-dependent p21 signaling. Cell Signal.

[8] Bolignano D, Mattace-Raso F, Sijbrands EJ, Zoccali C (2014). The aging kidney revisited: a systematic review. Ageing Res Rev.

[9] Corsonello A, Aucella F, Pedone C, Antonelli-Incalzi R (2017). Chronic kidney disease: a likely underestimated component of multimorbidity in older patients with chronic obstructive pulmonary disease. Geriatr Gerontol Int.

[10] Glassock RJ, Rule AD (2012). The implications of anatomical and functional changes of the aging kidney: with an emphasis on the glomeruli. Kidney Int.

[11] Lindeman RD, Tobin J, Shock NW (1985). Longitudinal studies on the rate of decline in renal function with age. J Am Geriatr Soc.

[12] Wetzels JF, Kiemeney LA, Swinkels DW, Willems HL, den Heijer M (2007). Age- and gender-specific reference values of estimated GFR in Caucasians: the Nijmegen Biomedical Study. Kidney Int.

[13] Glassock RJ, Denic A, Rule AD (2017). The conundrums of chronic kidney disease and aging. J Nephrol.

[14] Denic A, Mathew J, Lerman LO (2017). Single-nephron glomerular filtration rate in healthy adults. N Engl J Med.

[15] Esposito C, Dal Canton A (2010). Functional changes in the aging kidney. J Nephrol.

[16] Musso CG, Macias Nunez JF, Oreopoulos DG (2007). Physiological similarities and differences between renal aging and chronic renal disease. J Nephrol.

[17] Ershler WB, Sheng S, McKelvey J (2005). Serum erythropoietin and aging: a longitudinal analysis. J Am Geriatr Soc.

[18] Musso CG, Oreopoulos DG (2011). Aging and physiological changes of the kidneys including changes in glomerular filtration rate. Nephron Physiol.

[19] Schuck O, Nadvornikova H (1987). Short acidification test and its interpretation with respect to age. Nephron.

[20] KDIGO 2012 Clinical Practice Guideline for The Evaluation and Management of Chronic Kidney Disease (2013). Chapter 1: Definition and classification of CKD. Kidney Int Suppl.

[21] Hemmelgarn BR, Zhang J, Manns BJ (2006). Progression of kidney dysfunction in the community-dwelling elderly. Kidney Int.

[22] Obi Y, Kimura T, Nagasawa Y (2010). Impact of age and overt proteinuria on outcomes of stage 3–5 chronic kidney disease in a referred cohort. Clin J Am Soc Nephrol.

[23] Okabayashi Y, Tsuboi N, Haruhara K (2016). Reduction of proteinuria by therapeutic intervention improves the renal outcome of elderly patients with IgA nephropathy. Clin Exp Nephrol.

[24] O’Hare AM, Hotchkiss JR, Kurella Tamura M (2014). Interpreting treatment effects from clinical trials in the context of real-world risk information: end-stage renal disease prevention in older adults. JAMA Intern Med.

[25] Formiga F, Ferrer A, Sanz H (2013). Patterns of comorbidity and multimorbidity in the oldest old: the Octabaix study. Eur J Intern Med.

[26] Zemedikun DT, Gray LJ, Khunti K, Davies MJ, Dhalwani NN (2018). Patterns of multimorbidity in middle-aged and older adults: an analysis of the UK Biobank Data. Mayo Clin Proc.

[27] Bulcun E, Ekici M, Ekici A, Cimen DA, Kisa U (2015). Microalbuminuria in obstructive sleep apnea syndrome. Sleep Breath.

[28] Casanova C, de Torres JP, Navarro J (2010). Microalbuminuria and hypoxemia in patients with chronic obstructive pulmonary disease. Am J Respir Crit Care Med.

[29] Corsonello A, Pedone C, Corica F (2005). Concealed renal insufficiency and adverse drug reactions in elderly hospitalized patients. Arch Intern Med.

[30] Incalzi RA, Corsonello A, Pedone C (2010). Chronic renal failure: a neglected comorbidity of COPD. Chest.

[31] Chawla LS, Amdur RL, Amodeo S, Kimmel PL, Palant CE (2011). The severity of acute kidney injury predicts progression to chronic kidney disease. Kidney Int.

[32] Ishani A, Xue JL, Himmelfarb J (2009). Acute kidney injury increases risk of ESRD among elderly. J Am Soc Nephrol.

[33] Bucaloiu ID, Kirchner HL, Norfolk ER, Hartle JE, Perkins RM (2012). Increased risk of death and de novo chronic kidney disease following reversible acute kidney injury. Kidney Int.

[34] Mehran R, Aymong ED, Nikolsky E (2004). A simple risk score for prediction of contrast-induced nephropathy after percutaneous coronary intervention: development and initial validation. J Am Coll Cardiol.

[35] Yancy CW, Lopatin M, Stevenson LW (2006). Clinical presentation, management, and in-hospital outcomes of patients admitted with acute decompensated heart failure with preserved systolic function: a report from the Acute Decompensated Heart Failure National Registry (ADHERE) Database. J Am Coll Cardiol.

[36] Rusinaru D, Buiciuc O, Houpe D, Tribouilloy C (2011). Renal function and long-term survival after hospital discharge in heart failure with preserved ejection fraction. Int J Cardiol.

[37] Ravanan R, Spiro JR, Mathieson PW, Smith RM (2007). Impact of diabetes on haemoglobin levels in renal disease. Diabetologia.

[38] K/DOQI Clinical (2003). practice guidelines for bone metabolism and disease in chronic kidney disease. Am J Kidney Dis.

[39] Levey AS, Stevens LA, Schmid CH (2009). A new equation to estimate glomerular filtration rate. Ann Intern Med.

[40] Schaeffner ES, Ebert N, Delanaye P (2012). Two novel equations to estimate kidney function in persons aged 70years or older. Ann Intern Med.

[41] Pottel H, Hoste L, Dubourg L (2016). An estimated glomerular filtration rate equation for the full age spectrum. Nephrol Dial Transpl.

[42] Bjork J, Grubb A, Gudnason V (2018). Comparison of glomerular filtration rate estimating equations derived from creatinine and cystatin C: validation in the Age, Gene/Environment Susceptibility-Reykjavik elderly cohort. Nephrol Dial Transpl.

[43] Losito A, Zampi I, Pittavini L, Zampi E (2017). Association of reduced kidney function with cardiovascular disease and mortality in elderly patients: comparison between the new Berlin initiative study (BIS1) and the MDRD study equations. J Nephrol.

[44] Raman M, Green D, Middleton RJ, Kalra PA (2018). Comparing the impact of older age on outcome in chronic kidney disease of different etiologies: a prospective cohort study. J Nephrol.

[45] Stacul F, van der Molen AJ, Reimer P (2011). Contrast induced nephropathy: updated ESUR Contrast Media Safety Committee guidelines. Eur Radiol.

[46] Palevsky PM, Liu KD, Brophy PD (2013). KDOQI US commentary on the 2012 KDIGO clinical practice guideline for acute kidney injury. Am J Kidney Dis.

[47] Spence JD, Urquhart BL, Bang H (2016). Effect of renal impairment on atherosclerosis: only partially mediated by homocysteine. Nephrol Dial Transpl.

[48] Perna AF, Ingrosso D (2012). Low hydrogen sulphide and chronic kidney disease: a dangerous liaison. Nephrol Dial Transpl.

[49] Soni R, Horowitz B, Unruh M (2013). Immunization in end-stage renal disease: opportunity to improve outcomes. Semin Dial.

[50] Hall RK, Haines C, Gorbatkin SM (2016). Incorporating geriatric assessment into a nephrology clinic: preliminary data from two models of care. J Am Geriatr Soc.

[51] Vendemia F, Camerun JS, Oreoupulos DG, Macias-Nunez JF (2008). The diagnosis of renal diseases in elderly patients. What role is there for biopsy?. The aging kidney in health and disease.

[52] Aucella F, Guida CC, Lauriola V, Vergura M (2010). How to assess renal function in the geriatric population. J Nephrol.

[53] Corsonello A, Pedone C, Bandinelli S, Ferrucci L, Antonelli Incalzi R (2017). Agreement between chronic kidney disease epidemiological collaboration and berlin initiative study equations for estimating glomerular filtration rate in older people: the Invecchiare in Chianti (Aging in Chianti Region) study. Geriatr Gerontol Int.

[54] KDIGO CKD Work Group (2013). KDIGO 2012 clinical practice guideline for the evaluation and management of chronic kidney disease. Kidney Int Suppl.

[55] Farrington K, Covic A, Nistor I (2017). Clinical Practice Guideline on management of older patients with chronic kidney disease stage 3b or higher (eGFR<45ml/min/1.73m^2^): a summary document from the European Renal Best Practice Group. Nephrol Dial Transpl.

[56] Glassock RJ, Con (2014). Thresholds to define chronic kidney disease should not be age dependent. Nephrol Dial Transpl.

[57] Glassock R, Delanaye P, El Nahas M (2015). An age-calibrated classification of chronic kidney disease. JAMA.

[58] Conte G, Minutolo R, De Nicola L, Pro (2014). Thresholds to define chronic kidney disease should not be age-dependent. Nephrol Dial Transpl.

[59] John R, Webb M, Young A, Stevens PE (2004). Unreferred chronic kidney disease: a longitudinal study. Am J Kidney Dis.

[60] Minutolo R, Lapi F, Chiodini P (2014). Risk of ESRD and death in patients with CKD not referred to a nephrologist: a 7-year prospective study. Clin J Am Soc Nephrol.

[61] McClure M, Jorna T, Wilkinson L, Taylor J (2017). Elderly patients with chronic kidney disease: do they really need referral to the nephrology clinic?. Clin Kidney J.

[62] De Nicola L, Minutolo R, Chiodini P (2012). The effect of increasing age on the prognosis of non-dialysis patients with chronic kidney disease receiving stable nephrology care. Kidney Int.

[63] Hoefield RA, Kalra PA, Baker P (2010). Factors associated with kidney disease progression and mortality in a referred CKD population. Am J Kidney Dis.

[64] O’Hare AM, Choi AI, Bertenthal D (2007). Age affects outcomes in chronic kidney disease. J Am Soc Nephrol.

[65] Borrelli S, Leonardis D, Minutolo R (2015). Epidemiology of CKD regression in patients under nephrology care. PLoS One.

[66] Baek SH, Ahn S, Lee SW (2015). Outcomes of predialysis nephrology care in elderly patients beginning to undergo dialysis. PLoS One.

[67] Corsonello A, Fusco S, Bustacchini S (2016). Special considerations for the treatment of chronic kidney disease in the elderly. Expert Rev Clin Pharmacol.

[68] Pilotto A, Ferrucci L, Franceschi M (2008). Development and validation of a multidimensional prognostic index for one-year mortality from comprehensive geriatric assessment in hospitalized older patients. Rejuvenation Res.

[69] Sy J, Johansen KL (2017). The impact of frailty on outcomes in dialysis. Curr Opin Nephrol Hypertens.

[70] Antonelli Incalzi R, Aucella F, Leosco D, Brunori G, Dalmartello M, Paolisso G (2015). Assessing nephrological competence among geriatricians: a proof of concept internet survey. PLoS One.

[71] Aucella F, Brunori G, Dalmartello M (2016). Assessment of the geriatric competence and perceived needs of Italian nephrologists: an internet survey. J Nephrol.

[72] Levey AS, Inker LA, Coresh J (2015). Chronic kidney disease in older people. JAMA.

[73] Jones CA, Francis ME, Eberhardt MS (2002). Microalbuminuria in the US population: third National Health and Nutrition Examination Survey. Am J Kidney Dis.

[74] Viazzi F, Leoncini G, Ratto E (2007). Mild hyperuricemia and subclinical renal damage in untreated primary hypertension. Am J Hypertens.

[75] Pearce JD, Edwards MS, Craven TE (2005). Renal duplex parameters, blood pressure, and renal function in elderly people. Am J Kidney Dis.

[76] Petrucci I, Samoni S, Meola M (2016). Clinical scenarios in chronic kidney disease: parenchymal chronic renal diseases—part 2. Contrib Nephrol.

[77] Zanoli L, Romano G, Romano M (2014). Renal function and ultrasound imaging in elderly subjects. Sci World J.

[78] Briganti EM, Dowling J, Finlay M (2001). The incidence of biopsy-proven glomerulonephritis in Australia. Nephrol Dial Transpl.

[79] Pfister M, Jakob S, Frey FJ, Niederer U, Schmidt M, Marti HP (1999). Judgment analysis in clinical nephrology. Am J Kidney Dis.

[80] Kitterer D, Gurzing K, Segerer S (2015). Diagnostic impact of percutaneous renal biopsy. Clin Nephrol.

[81] Harmankaya O, Okuturlar Y, Kocoglu H (2015). Renal biopsy in the elderly: a single-center experience. Int Urol Nephrol.

[82] Kohli HS, Jairam A, Bhat A (2006). Safety of kidney biopsy in elderly: a prospective study. Int Urol Nephrol.

[83] Rollino C, Ferro M, Beltrame G (2014). Renal biopsy in patients over 75: 131 cases. Clin Nephrol.

[84] Haas M, Spargo BH, Wit EJ, Meehan SM (2000). Etiologies and outcome of acute renal insufficiency in older adults: a renal biopsy study of 259 cases. Am J Kidney Dis.

[85] Mazzucco G, Bertani T, Fortunato M (2002). Different patterns of renal damage in type 2 diabetes mellitus: a multicentric study on 393 biopsies. Am J Kidney Dis.

[86] Vendemia F, Gesualdo L, Schena FP, D’Amico G, Renal Immunopathology Study Group of the Italian Society of N (2001). Epidemiology of primary glomerulonephritis in the elderly. Report from the Italian Registry of Renal Biopsy. J Nephrol.

[87] Heaf J (2004). The Danish Renal Biopsy Register. Kidney Int.

[88] Simon P, Ramee MP, Boulahrouz R (2004). Epidemiologic data of primary glomerular diseases in western France. Kidney Int.

[89] Haas M, Spargo BH, Wit EJC, Meehan SM (2000). Etiologies and outcome of acute renal insufficiency in older adults: a renal biopsy study of 259 cases. Am J Kidney Dis.

[90] Ponticelli C, Passerini P, Como G (1993). Primary nephrotic syndrome in the elderly. Contrib Nephrol.

[91] Chen Y, Li P, Cui CL, Yuan AH, Zhang K, Yu C (2016). Biopsy-proven kidney diseases in the elderly: clinical characteristics, renal histopathological spectrum and prognostic factors. J Int Med Res.

